# Serotonin Receptors in Myocardial Infarction: Friend or Foe?

**DOI:** 10.1021/acschemneuro.4c00031

**Published:** 2024-04-04

**Authors:** F.S. Bahr, M. Ricke-Hoch, E. Ponimaskin, F.E. Müller

**Affiliations:** † Cellular Neurophysiology, Hannover Medical School, 30625 Hannover, Germany; ‡ Cardiology and Angiology, Hannover Medical School, 30625 Hannover, Germany

**Keywords:** Myocardial infarction, Serotonin receptors, 5-HT system, Cardiovascular system, Serotonin reuptake inhibitors, Cardioprotection

## Abstract

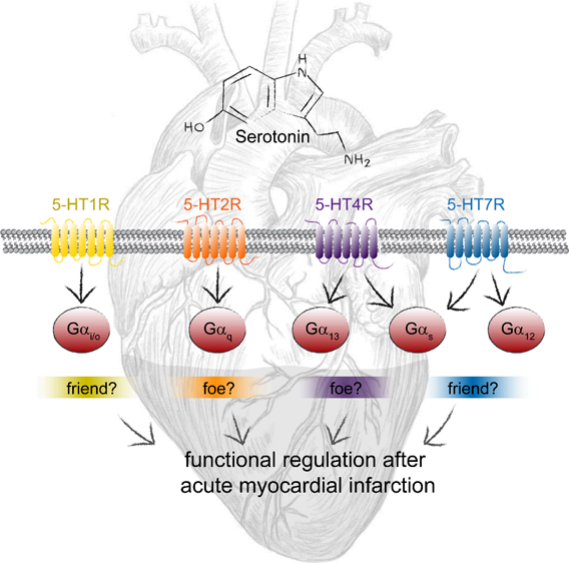

Acute myocardial infarction (AMI) is one of the leading causes of death worldwide and treatment costs pose a major burden on the global health care system. Despite the variety of treatment options, individual recovery can be still poor and the mortality rate, especially in the first few years after the event, remains high. Therefore, intense research is currently focused on identifying novel target molecules to improve the outcome following AMI. One of the potentially interesting targets is the serotonergic system (5-HT system), not at least because of its connection to mental disorders. It is known that patients suffering from AMI have an increased risk of developing depression and *vice versa*. This implicates that the 5-HT system can be affected in response to AMI and might thus represent a target structure for patients’ treatment. This review aims to highlight the importance of the 5-HT system after AMI by describing the role of individual serotonin receptors (5-HTR) in the regulation of physiological and pathophysiological responses. It particularly focuses on the signaling pathways of the serotonin receptors 1, 2, 4, and 7, which are expressed in the cardiovascular system, during disease onset, and the following remodeling process. This overview also emphasizes the importance of the 5-HT system in AMI etiology and highlights 5-HTRs as potential treatment targets.

## Introduction

Globally, one of the main causes of death is acute myocardial infarction (AMI) and ensuing heart failure.^1^ The morbidity and mortality of AMI is still high in western societies with nearly 550,000 first episodes and 200,000 recurrent episodes of AMI emerging annually in the US.^2^

AMI occurs when the blood supply to defined regions of the myocardium is interrupted, for example, by vulnerable atherosclerotic plaque rupture or coronary artery occlusion.^3^ The restriction in blood supply leads to ischemia and hypoxia in the affected parts of the myocardium, resulting in loss of cardiac tissue due to cardiomyocyte death and tissue necrosis. The induction of cardiac cell death triggers a cascade of molecular processes that promote wound healing and structural cardiac remodeling. Here, monocytes and macrophages act as key modulators in the wound repair process through the clearance of necrotic cells and the release of cytokines and chemokines, proteases, growth factors, and oxygen-derived free-radicals.^4,5^

After initial infarct extension, a healing process is started that involves resorption of necrotic tissue and formation of the infarct scar that replaces the cardiac tissue. The remodeling process occurs after myocardial injury and includes loss of cardiomyocytes and capillaries, hypertrophy, and fibrosis in the surviving myocardium. In addition, remodeling takes place also in the infarct scar and the borderzone that can lead to infarct scar thinning and elongation which both increase the risk of fatal left ventricle (LV) rupture and further contribute to remodeling by impaired ventricular geometry.^3^ Within the first 2 weeks after injury, the healing in infarcted hearts is dominated by inflammatory processes. After the initial ischemia, dead cells induce an inflammatory response characterized by the activation of the complement system and the release of reactive oxygen species and paracrine factors from inflammatory or endothelial cells promoting the recruitment of high numbers of neutrophils to the site of injury. In addition, the apoptosis of neutrophils stimulates the migration of large numbers of macrophages into the infarcted tissue that eliminate dead cells and secrete cytokines and chemokines. After approximately 2 days, the acute inflammation ceases and the proliferative phase starts, where inflammatory cells release factors that promote angiogenesis and fibroblast proliferation. Furthermore, these factors also promote the differentiation of fibroblasts into myofibroblasts that produce extracellular matrix to form the infarct scar. After the first week, the transition to the maturation phase is initiated by the formation of a collagen-rich scar with multiple vessels surrounded by pericytes.^6^ During the course of disease, the heart may experience additional pathological insults such as dilation, arrhythmias, increased end-diastolic and end-systolic volume, increased wall tension, and a reduced ejection fraction (EF), all of that can promote the transition to heart failure. The regulation of the inflammatory response seems to be a critical factor in infarct healing and remodeling. It is therefore not surprising that it has been explored as a therapeutic target to prevent functional decline and is the focus of current research.^7−9^

The pathophysiological impact of AMI extends beyond the heart, affecting organs of the immune system and the central nervous system (CNS).^10^ It has been shown that patients with heart failure are frequently affected by major depressive disorder (MDD; prevalence 20–35%).^11^ Inversely, people with severe psychiatric disorders, such as MDD, have an increased risk of developing cardiovascular disease, associated with an increase in morbidity and mortality.^12,13^ This increased incidence is aggravated by worsened cardiovascular prognosis, and risk of death is significantly higher for depressed patients compared to nondepressed patients in the first 18 month after AMI. Thus, clinical depression has been identified as an independent risk factor for heart disease.^14−18^

Although the precise interrelation between MDD and AMI has not yet been established, dysregulation of the serotonergic system (5-HT system) might be involved in the etiology of both AMI and MDD. Consequently, selective serotonin reuptake inhibitors (SSRIs) used in the treatment for MDD have been investigated as a protective therapy against AMI, and in many cases, such treatment has produced a positive outcome.^19^

Serotonin, or 5-hydroxytryptamine (5-HT), was discovered more than 70 years ago for its function to increase gut contractility.^20^ Since then, many more functions have been described, including its importance in the CNS for regulating cognitive functions, mood stabilization, sleeping, and feeding behavior. Noteworthy, all these processes are governed by only about 5% of the body’s own 5-HT, which is produced in the raphe nuclei of the brain stem. The residual 95% of 5-HT is synthesized in the periphery, mainly by enterochromaffin cells in the gut, but also in other peripheral tissues including the heart.^21^ Of note, peripheral 5-HT has no effect on CNS functions because serotonin can hardly pass the intact blood-brain-barrier. Serotonin released from the enterochromaffin cells can be taken up by platelets, which are the main storage for the peripheral 5-HT. Via this route, 5-HT enters the cardiac capillaries and can act on local serotonin receptors, when released from platelets. More recent evidence suggests that 5-HT can be synthesized in small amounts by coronary endothelial cells as well as in human atria and mouse cardiomyocytes.^22,23^ Pönicke and colleagues detected tryptophan hydroxylase (TPH) and aromatic L-amino acid decarboxylase in mouse cardiac and human atrial preparations and also found monoamine oxidase crucial for 5-HT degradation. The physiological functions of 5-HT in the human cardiovascular system include vasoconstriction and platelet aggregation, and it exerts positive chronotropic and inotropic effects on the heart.

The serotonin transporters (5-HTT or SERT) are highly expressed on thrombocytes but might also represent a way for how 5-HT can enter cardiomyocytes. The 5-HTT is the target of SSRIs like fluoxetine, escitalopram, and sertraline, which represent common antidepressant drugs used in the treatment of MDD. While low-dose tricyclic antidepressants (TCAs), which do not target the serotonergic system, were associated with increased risk of major adverse cardiovascular events,^24^ the use of SSRIs is associated with fewer cardiovascular events in patients with depression.^19^ After AMI, elevated blood levels of 5-HT have been detected, most likely released from activated platelets. This increase of 5-HT levels can profoundly impact activation of 5-HT receptors (5-HTRs) expressed on the heart itself, but also on immune cells, which are invading into the infarcted region, and therefore mediate the initiated immune response. The protective effect of SSRI treatment in patients treated for MDD might be related to the inhibition of serotonin-mediated platelet activation.^25^ Additionally, long-term use of SSRIs is supposed to deplete platelets from 5-HT, which in the case of AMI can reduce the amount of released 5-HT to reduce myocardial injury. This process was linked to 5-HT-mediated enhanced neutrophil degranulation, which results in increased damage to the infarcted area and inflammation.^26^

In the following, we focus on the possible role of different 5-HTRs in cardiovascular diseases and especially in AMI.

## Main

### Serotonin Receptors in the Heart

**Figure 1:**
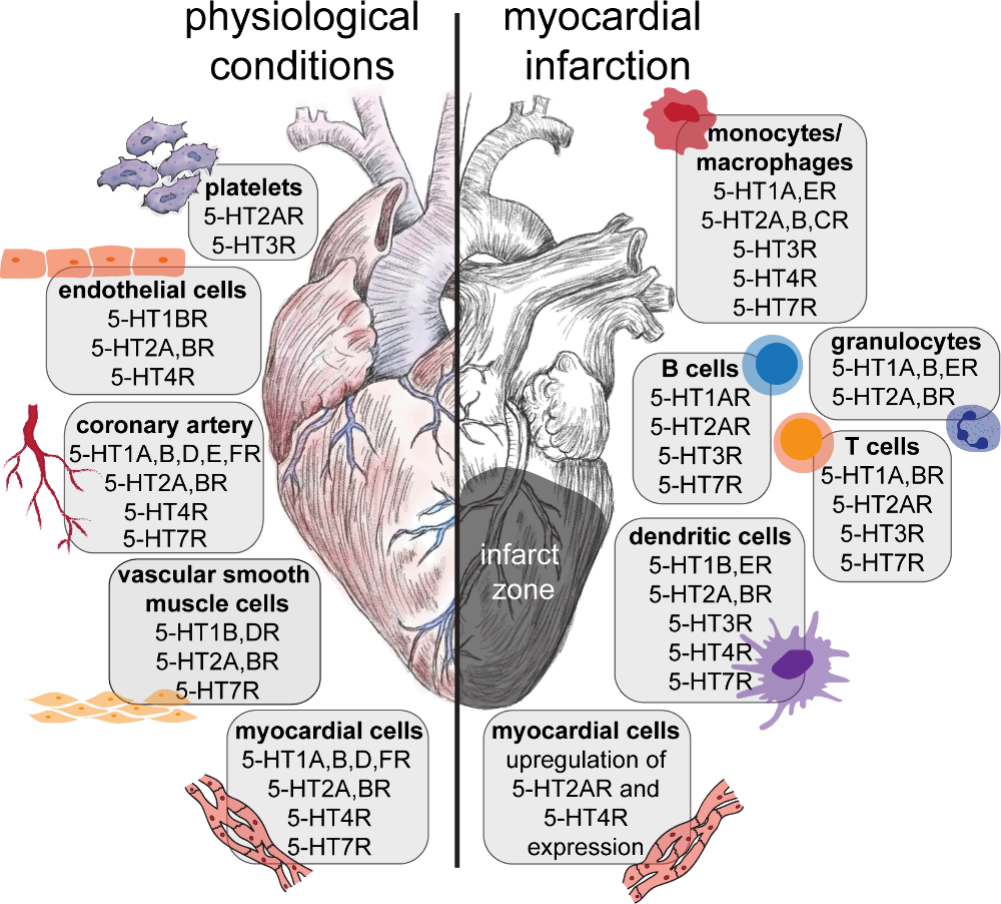
Expression of different 5-HTRs in the cardiovascular unit under physiological conditions (left side) and after AMI (right side), including relevant infiltrating immune cells. The group of myocardial cells comprise cardiomyocytes and fibroblasts of both atrium and ventricles.

The physiological effects of 5-HT on the heart are complex, and several studies even show contradictory results. For example, it was shown that 5-HT can both increase or decrease blood pressure, induce bradycardia or tachycardia, have local vasodilatory or vasoconstrictive effects, and stimulate mitogenesis in isolated cardiomyocytes.^27,28^ These various effects can be at least partly explained by 5-HT acting via different members of the serotonin receptor family (5-HT1R to 5-HT7R), four of which are expressed within the heart tissue (see Table 1 and Figure 1). These include the G protein-coupled receptors (GPCRs) 5-HT1R, 5-HT2R, 5-HT4R, and 5-HT7R. Of note, 5-HT can also influence cardiac function via the 5-HT3R, a ligand-gated ion channel, which is located on the vagus nerve endings in the heart and whose activation induces a short-term hypotension lowering the heart rate (i.e., von Bezold-Jarisch-like reflex).^27^

**Table 1:** Overview of Human 5-HTR Expression in the Cardiovascular System with Relevance to AMI^a^

5-HTR	G protein-coupling	Expression in the heart^(a)^	Expression on immune cells^(b)^	Cardiac physiological function and effects^(c)^	Role in AMI^(d)^	Refs
**1A**	Gαi/o	coronary arteries, atrium, and ventricles	mast cells (m PCR), monocytes (stim), macrophages (m IHC), natural killer cells (stim), T cells (+Northern blot), B cells (stim/flow), and eosinophils	lower heart rate and arterial blood pressure, vasodilation, and suppression of stress- and inflammation-induced tachycardiac response		(a) (29 and 30), (b) (31−38), and (c) (39−41)
**1B**	Gαi/o	vascular smooth muscle cells (+IHC), endothelial cells (IHC), coronary arteries (+pig PCR), atrium (+Western blot), and ventricles	dendritic cells, T cells (m PCR), and eosinophils	vasoconstriction		(a) (29, 30, 42, and 43), (b) (32, 44, and 45), and (c) (43)
**1D**	Gαi/o	vascular smooth muscle cells (rabbit PCR), coronary arteries, atrium, and ventricle		vasoconstriction		(a) (29, 30, 42, and 46) and (c) (47)
**1E**	Gαi/o	coronary arteries, not found in mice and rats, not expressed in guinea pig heart	monocytes, dendritic cells, and eosinophils			(a) (29 and 48) and (b) (32, 44, and 49)
**1F**	Gαi/o	coronary arteries, atrium, and ventricles				(a) (30)
**2A**	Gαq/11, PLC	vascular smooth muscle cells (+IHC), platelets (rat stim), endothelial cells, coronary arteries (+pig PCR), atrium, and ventricles	monocytes, macrophages (m PCR/IHC), dendritic cells, eosinophils, T cells (m PCR), and B cells (flow)	positively chronotropic, vasoconstriction	downregulated in the brain of patients after AMI, while upregulated in the heart; gene polymorphisms no suitable genetic marker for AMI; inhibition is cardioprotective: inhibition reduces thrombus formation and hypertrophic remodeling; inhibition improves cardiac LV function and angiogenesis	(a) (29, 30, 43, and 50−52), (b) (32, 38, 44, 45, 49, 53, and 54), (c) (30, 55, and 56), and (d) (57−69)
**2B**	Gαq/11, PLC	endothelial cells, vascular smooth muscle cells, coronary arteries (+pig PCR), cardiac fibroblasts (IHC, and m Southern Blot)	macrophages, dendritic cells, and eosinophils	ablation is lethal in mouse model, heart development, mitogenesis, and sustained hypertension		(a) (29, 50, and 70), (b) (32, 44, and 71), and (c) (72−80)
**2C**	Gαq/11, PLC		macrophages (m PCR)	hypertension		(b) (81) and (c) (82)
**4**	GαS/13	endothelial cells, atrium, ventricles, and coronary artery (pig PCR)	monocytes and dendritic cells	positively chronotropic and inotropic, upregulated in congestive heart failure	upregulated after AMI	(a) (50, 83, and 84), (b) (44 and 49), (c) (84 and 85), and (d) (84)
**7**	GαS/12	vascular smooth muscle cells, coronary arteries (+pig PCR), atrium, ventricles, and endothelial cells	monocytes, macrophages, dendritic cells, T cells (m PCR), and B cells (flow)	positively chronotropic, vasodilation, increase coronary blood flow	activation improved tissue remodeling after AMI	(a) (30, 50, and 86), (b) (38, 44, 45, 49, and 71), (c) (87−94), and (d) (95)

a Unless otherwise noted, human serotonin receptor expression was detected by PCR. Well-working antibodies against 5-HTRs are mostly not available. Symbol legend: + additional method; IHC: immunohistochemistry; flow: flow cytometry; stim: pharmacological treatment; and m: mouse.

## 5-HT1R

**Figure 2:**
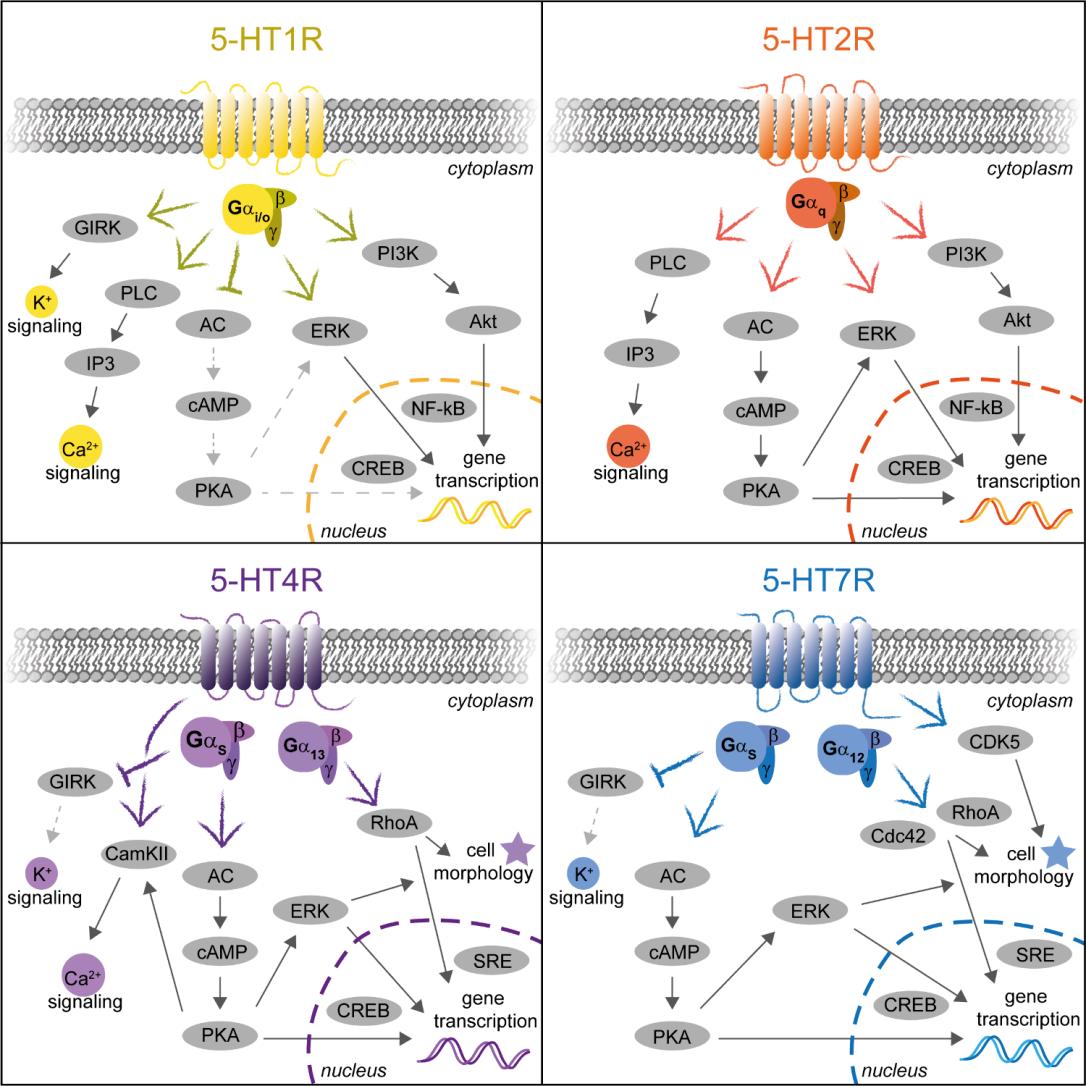
Main signaling pathways of 5-HT1R, 5-HT2R, 5-HT4R, and 5-HT7R.

The subfamily of 5-HT1Rs includes five receptor subtypes (5-HT1AR, 5-HT1BR, 5-HT1DR, 5-HT1ER, and 5-HT1FR), which share 40–63% sequence identity in humans. All these receptors are coupled to Gαi/Gαo proteins to inhibit the adenylyl cyclase (AC)-cyclic adenosine monophosphate (cAMP)-protein kinase A (PKA)-pathway (Figure 2). This is an interesting fact, as activation of the AC-cAMP pathway is the main signaling effector of β-adrenergic receptors, whose inhibition is a common treatment strategy for decades in many pathologies of the heart, including AMI.^96^ This intervention is, however, not without side-effects, and the use of β-adrenergic receptor blockers is continuously debated in context of new treatment strategies and drug developments.^97^ The beneficial effect of AC-cAMP inhibition seems fairly assured; maybe it is time to explore the targeting of another GPCR for its regulation?

5-HT1Rs were also shown to influence calcium signaling via the phospholipase C (PLC)-inositol 1,4,5-trisphosphate (IP3) axis and to activate the phosphoinositide-3-kinase (PI3K)-protein kinase B (PKB, or Akt) signaling pathway. Furthermore, 5-HT1R signaling regulates the mammalian target of rapamycin (mTOR) and mediates extracellular-signal regulated kinase/mitogen-activated protein kinase (ERK/MAPK) signaling to modulate nuclear factor “kappa-light-chain-enhancer” of activated B-cells (NF-kB).^98−100^ Changes in 5-HT1R-mediated signaling have been associated with anxiety and depressive disorders.^101^ These in turn are risk factors for cardiovascular diseases.^102^

One of the best studied members of this receptor subfamily is the 5-HT1AR, which was shown across studies and species to suppress stress- and inflammation-induced tachycardiac response upon activation.^41^ This response, however, is processed in the brain and not the heart itself. In the cardiovascular system, the 5-HT1AR is expressed in human coronary arteries, as well as the atrium and ventricles.^30^ It is also expressed on immune cells, including mast cells and macrophages, where it can modulate chemotaxis, adhesion, and phagocytosis.^33,34^

Pharmacological activation of the 5-HT1AR by agonists like flesinoxan and 8-hydroxy-2-(di-N-propylamino)tetralin (8-OH-DPAT) showed vasodilatory effects and lowered blood pressure and heart rate in rats.^40^ Moreover, administration of 8-OH-DPAT lowered body temperature and improved survival after transient cardiac arrest in rats in a different study. Hypothermia after cardiac arrest occurs spontaneously in small rodents and seems neuroprotective; however, 8-OH-DPAT treatment did improve neuronal survival and sensorimotor integration behavior after 7 days in this study.^39^

The 5-HT1BR is expressed on smooth muscle cells and endothelial cells of both veins and arteries where it can regulate the vascular tone, acting as a vasoconstrictor.^43^ Similarly, activation of the 5-HT1DR, expressed on smooth muscle cells, leads to contraction of the large coronary arteries in preparations from human hearts.^47^ The 5-HT1FR was found to be highly expressed in the human coronary arteries, epicardium, atrium, and ventricles, but its cardiovascular function remains unknown.^30^ In contrast to the above-mentioned receptors, the 5-HT1ER has not been found within the heart tissue of investigated species.^48,103^ It is however present in eosinophils and monocytes/macrophages and can thus be involved in modulation of inflammatory responses after AMI (see Figure 1).

## 5-HT2R

The 5-HT2R subfamily was first characterized in 1979^104^ and comprises three subgroups (5-HT2AR, 5-HT2BR, and 5-HT2CR), which share about 50% sequence homology.^99^ All 5-HT2Rs couple to the Gαq protein to activate the PLCβ signaling pathway (Figure 2). Independently from this canonical pathway, Gαq can activate p63RhoGEF, which provides a link between GPCRs and ras homolog family member A (RhoA) activation, PI3K, implicated in the regulation of the Akt pathway. Furthermore, direct binding of Gαq inhibits the cold-activated transient receptor potential cation channel subfamily M member 8 (TRPM8), responsible for abnormal cold sensation in inflammation.^105^ Recently, the activation of ERK/MAPK by Gαq-coupled receptors has also been described as a novel PLCβ-independent signaling axis that relies upon the interaction between this G protein and two novel effectors (protein kinase C ζ (PKCζ) and MEK5).^106^ Additionally, the association of Gαq with different regulatory proteins can modulate its effector coupling ability and, therefore, its signaling potential.

More specifically, the 5-HT2AR can regulate calcium dynamics and PKC activity.^107,108^ Moreover, both 5-HT2AR and 5-HT2BR can influence cyclic guanosine monophosphate (cGMP) kinetics via phospholipase A2 and nitric oxide synthase activation.^109−111^

In contrast to the 5-HT2R family members A and B, which will be described in the following sections in more detail, the 5-HT2CR shows strong expression in various brain regions, while its expression was not detected in the body periphery.^112^ However, the CNS-expressed 5-HT2CRs were shown to participate in cardiovascular regulation because receptor activation induced hypertension and was necessary to increase blood pressure in rats.^82^

### 5-HT2AR in the Cardiovascular System

The 5-HT2AR was initially detected in the rat brain.^104^ Later, its expression was confirmed in distinct brain regions as well as in human smooth muscle cells, cardiac muscle cells, and platelets (see Figure 1).^29,42,52^ Interestingly, the polymorphism T102C within exon 1 of the 5-HT2AR gene was proposed to act as a genetic marker for AMI.^113^ However, follow-up studies could not find any evidence connecting this polymorphism with AMI.^58−60^ Further attempts to correlate polymorphisms of the 5-HTT and 5-HT2AR with susceptibility for AMI showed no influence on occurrence of adverse cardiac events.^59^ Schins et al. detected less 5-HT2AR in the brains of AMI-patients compared to non-AMI controls. Interestingly, depressed AMI-patients showed increased radioligand binding to 5-HT2AR compared to nondepressed AMI-patients.^57^ More recently, Williams et al. demonstrated that depressed cardiovascular patients have higher 5-HT2AR density on platelets and significantly higher incidence of major and minor cardiac adverse events.^114^ Moreover, 5-HT2AR-mediated signaling can regulate the vascular tone leading to vasoconstriction.^30,55,56^ Enhanced 5-HT2AR expression was detected in arteries compared to veins and in the left coronary artery compared to the right coronary artery in rats and swine, respectively.^52,115^ Interestingly, although AMI has been shown to facilitate transition of cardiac fibroblasts into myofibroblasts resulting in increased expression of SERT and TPH1, it does not result in any changes in 5-HT2AR expression in these myofibroblasts.^116^

**Figure 3:**
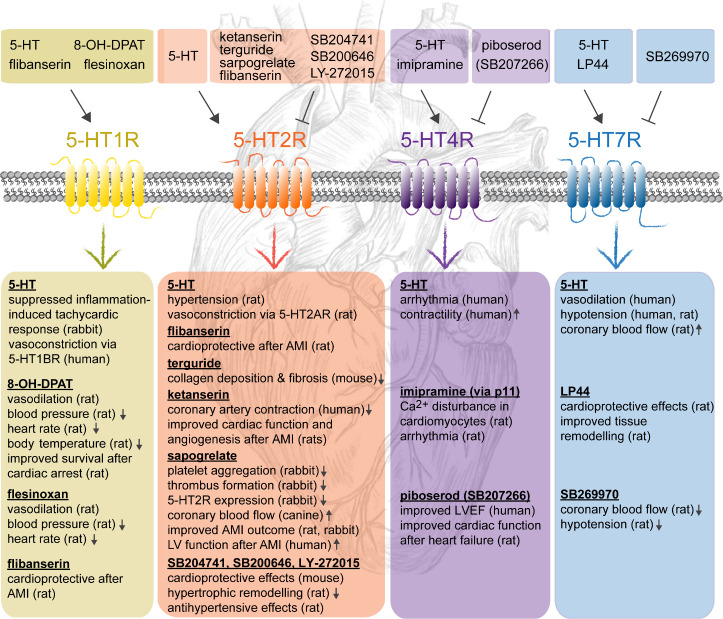
Effects of activators and inhibitors of the 5-HT1R, 5-HT2R, 5-HT4R, and 5-HT7R on the cardiovascular system under physiological conditions and in cardiac disease.

Pharmacological inhibition of 5-HT2AR activity showed cardioprotective effects with repressed hypertrophic remodeling. Such treatment leads to reduced receptor expression and might correlate with the degree of hypertrophy (see Figure 3).^63,117^ Treatment with the receptor inhibitor ketanserin led to reduced left coronary artery contraction in response to 5-HT stimulation, rendering the 5-HT2AR responsible for this reaction.^52,115^ Ketanserin was further shown to improve cardiac function and angiogenesis in ischemic myocardial tissue after AMI in rats and was suggested for therapeutic treatment after AMI in humans.^65,66^ In the study by Yu et al., ketanserin (0.3 mg/kg daily) was orally administered starting 2 weeks before the left coronary artery ligation and long-term treatment effects were assessed up to three month later, suggesting chronic treatment.

Repeated balloon-injury to the rabbit femoral artery showed less platelet aggregation and thrombus formation after treatment with the selective 5-HT2AR antagonist sarpogrelate hydrochloride.^61^ In addition, chronic treatment with sarpogrelate hydrochloride reduced receptor expression and activated signaling over 5-HT1BR and nitric oxide enhancing coronary blood flow.^118−120^ Sarpogrelate treatment was also beneficial in rat and rabbit models of AMI: In rats, sarpogrelate treatment (5 mg/kg daily) lowered mortality, reduced infarct size, and attenuated electrocardiographic changes assessed 3 weeks after AMI. These results were consistent in two experimental designs, starting treatment 3 days before or 1 h post-AMI-induction.^62^ In a rat model of ischemia/reperfusion injury, sarpogrelate (4 mg/kg) administered before or during reperfusion improved recovery of cardiac functions, reduced infarct size, preserved myocardial adenosine triphosphate (ATP) levels, and reduced apoptosis.^64^ Intravenous infusion for 1 h with sarpogrelate (10 mg/kg) before or after ischemia and reperfusion reduced infarct size and prevented elevation of 5-HT levels after 2 days in rabbits.^121^ In mice, oral administration of sarpogrelate (5 mg/kg) for 8 weeks after transverse aortic constriction suppressed cardiac hypertrophy and prevented systolic dysfunction. However, this more recent study suggests that these effects might occur both 5-HT2AR-dependent and -independent through inhibition of the ERK1/2 signaling pathway.^63^

Moreover, a combination antiplatelet-aggregation therapy with sarpogrelate treatment improved LV systolic function in patients 6 month after AMI.^122^ To further enhance treatment efficiency and provide alternatives for patients being resistant to currently used drugs, new 5-HT2AR antagonists are being synthesized and investigated for their ability to suppress platelet aggregation.^123^ One example is flibanserin, a drug which acts as agonist for 5-HT1AR and antagonist of 5-HT2AR. It is already approved for the treatment of hypoactive sexual desire disorder. Treatment with flibanserin evoked strong cardioprotective effects in a dose-dependent manner in an isoproterenol-induced model of AMI in rats.^69^ In this study, flibanserin treatment lowered heart rate, preserved architecture of myocardial fibers, and reduced 5-HT2AR expression in the cardiac tissue. However, flibanserin was given orally (45 mg/kg) for 4 weeks prior to AMI-induction, rather as a protective measure than post-AMI treatment, and the end-point of this study was only 1 day post AMI-induction. Long-term effects on cardiac tissue and stress markers should be investigated in the future.

These studies emphasize the role of 5-HT2AR activity in different cardiovascular diseases rendering this receptor a promising target for AMI treatment.

### The 5-HT2BR in the Cardiovascular System

This receptor was first identified in the rat fundus and later named 5-HT2BR.^29,42,124,125^ It is expressed in various regions of the CNS as well as in blood vessels, liver, heart, ovaries, lung, skeletal muscle, kidney, trachea, testis, small intestine, uterus, placenta, prostate, and pancreas.^42,125^ Also, cardiac fibroblasts and myofibroblasts express the 5-HT2BR.^70^ In addition, immune cells including macrophages, eosinophils, and dendritic cells express this receptor subtype.^32,44,71^ The 5-HT2BR modulates cell proliferation and contraction of muscle cells and is involved in pulmonary arterial hypertension, vascular hypertension, fibrosis, and valvular heart disease.^126^

The 5-HT2BR is a crucial regulator of heart development. Ablation of 5-HT2BR in a knockout mouse model led to embryonic and neonatal death due to cardiac defects.^127^ In line with this observation, overexpression of the 5-HT2BR in mouse hearts led to hypertrophic cardiomyopathy and excessive mitochondrial proliferation.^72^ Receptor activation or overexpression was further shown to lead to adverse remodeling with more collagen formation and reduced cardiac functions, while treatment with antagonists terguride and SB204741 reduced collagen deposition and fibrosis.^73−75,128^ Interestingly, the important female sex hormone estrogen can prevent cardiomyocyte death by binding to an estrogen responsive element, which regulates 5-HT2BR transcription. This cardiomyocyte protection is abolished by glucocorticoids, the main stress hormones, and therefore important in the context of women exposed to stress suffering from a heart attack.^129^

Under physiological conditions, regulation of blood pressure occurs via 5-HT2AR signaling, whose activation induces vasoconstriction.^56^ However, in a deoxycorticosterone acetate (DOCA)-salt-hypertensive rat model, which resembles human volume-overload induced chronic hypertension, contractions insensitive to the 5-HT2AR antagonist ketanserin were detected, together with increased 5-HT2BR expression. Treatment with the selective 5-HT2BR antagonists SB204741, SB200646, and LY-272015 showed a strong antihypertensive effect.^76−80^ This suggests that 5-HT2BR signaling is involved in development and maintenance of high blood pressure in DOCA salt-hypertensive rats and might thus be an important component for control of blood pressure regulation, essential after AMI.^130^

Taken together, the 5-HT2BR plays a fundamental role for heart functions, and modulation of receptor activity represents a promising therapeutic approach.

## 5-HT4R

The 5-HT4R was first identified in 1988 in mouse colliculi neurons,^131^ and its expression was then confirmed across various tissues and species. Among others, it is highly expressed in the human CNS, especially in the hippocampus, basal ganglia, and cortex^132^ but also in peripheral tissues including the gastrointestinal (GI)-tract, bladder, adrenal glands, intestine, and the heart.^133^ Within the human heart, 5-HT4R expression was shown in cardiomyocytes for both atrium and ventricles (see Figure 1).^83^ While in the CNS, its functions have been linked to anxiety, cognition, and memory,^134^ the 5-HT4R can directly influence cardiac contractility.^84^

At least 11 splice variants have been described in humans, with the 5-HT4bR being the most predominant isoform.^135,136^ In the human heart, these splice variants are differentially expressed: In the atrium 5-HT4aR, 5-HT4bR, 5-HT4cR, 5-HT4gR, 5-HT4iR, and 5-HT4nR isoforms were found, while in the ventricle 5-HT4aR, 5-HT4bR, 5-HT4gR, and 5-HT4iR isoforms were detected.^137^ All these isoforms share the ability to stimulate cAMP production via activation of the GαS signaling pathway (Figure 2). The 5-HT4aR additionally acts via the Gα13-RhoA signaling axis to modulate activity of small GTPases of the Rho family and gene transcription and was also shown to trigger an increase in intracellular [Ca^2+^]. Interestingly, the 5-HT4bR can induce GαS as well as Gαi signaling.^138,139^ To study the importance of 5-HT4R-induced GαS signaling and therewith increased cAMP levels in cardiac tissue, the effects of selective phosphodiesterase (PDE) enzymes, responsible for degradation of cAMP, were investigated after AMI in mice. This study suggests that inhibition of PDE enzymes is cardioprotective through regulation of neutrophil inflammation and microvascular obstruction.^140^ Therefore, in case 5-HT4R signaling should be a target for treatment after AMI, biased agonists and antagonists would be desirable to exclusively modulate the pathway of interest (i.e., modulation of cAMP levels). For example, RS67333 and prucalopride have been identified to induce cAMP production, while both inhibit IP3 formation.^141^

Of note, effects of selective modulation of 5-HT4Rs have prevalently been studied in chronic heart failure models rather than in AMI. An upregulation of 5-HT4bR-mRNA levels was reported in human ventricles after congestive heart failure.^85^ In this study, 5-HT application caused arrhythmias, whose generation could be blocked by 5-HT4R antagonists, therewith indicating therapeutic potential of this receptor. In addition, treatment with 5-HT4R-antagonist SB207266 improved *in vivo* cardiac function after congestive heart failure in rats. The rats received SB207266 (0.5 mg/kg daily) through mini-osmotic pumps for 6 weeks after post-AMI heart failure, which improved diastolic function, reduced heart and lung weight. Possible mechanisms suggested by the authors include decreased GαS-cAMP signaling, reducing the tissue energy consumption, alterations in the Ca^2+^ homeostasis, and Gα13-RhoA signaling implicated in cardiac hypertrophy.^142^ In humans, treatment with 5-HT4R-antagonist piboserod (80 mg/24 weeks) slightly improved left ventricular ejection fraction (LVEF), but neither other parameters nor overall quality of life.^143^

It has been shown that several antidepressants can elicit cardiotoxicity, and a study by Meschin and co-workers proposed that this effect could be explained by drug-induced modulation of 5-HT4R-mediated signaling. Antidepressants like imipramine enhance p11 expression, which tempers with 5-HT4R signaling, leading to deleterious Ca^2+^ disturbances in rat ventricular cardiomyocytes, promoting arrhythmias. Supporting this view, it has been shown that activation of cardiac 5-HT4R is associated with diastolic Ca^2+^ waves.^144^ For a recent review on 5-HT4R in cardiac health and disease see Neumann et al., 2023.^137^ Overall, the 5-HT4R represents an interesting target for treatment after AMI. Evidence suggests rather that inhibition of this receptor might promote beneficial effects.

## 5-HT7R

The latest identified member of the serotonin receptor family, 5-HT7R, was described and cloned in different species only in 1993. This receptor is highly expressed in the brain, coronary arteries (see Figure 1), GI-tract, liver, spleen, and on smooth muscle cells.^86,145−147^ In humans, three splice variants (5-HT7aR, 5-HT7bR, and 5-HT7dR) are known, which differ in their carboxyl-terminus amino acid sequence.^148^ The canonical signaling cascade of the 5-HT7R includes receptor-mediated activation of Gαs proteins (Figure 2).^86,146^ In addition, we have identified a noncanonical pathway via Gα12, leading to activation of the small GTPase cell division control protein 42 (Cdc42).^149,150^ More recently, we also described that 5-HT7R can activate the cyclin-dependent kinase 5 (CDK5) in a G protein-independent manner.^151^ Noteworthy, this receptor possesses a remarkable constitutive activity.^152,153^

### 5-HT7R and the Cardiovascular System

It has been shown that 5-HT has a vasodilative effect, which is mainly mediated via activation of vascular 5-HT7R, and this effect is more pronounced in veins than in arteries.^87,89−91^ Serotonin can also elicit upregulation of coronary blood flow in a 5-HT7R-dependent manner. This effect can therefore be pharmacologically achieved by activation of the 5-HT7R. On the contrary, application of the selective antagonist SB269970 reduced coronary flow.^88,89,92,94^ Additionally, Gonzalez-Pons et al. suggested that parallel inhibition of the 5-HT7R activity with 5-HT2AR activation leads to maximally increased contraction in veins.^154^ One recent study investigated the influence of the 5-HT7R agonist LP44 in rats suffering from isoproterenol-induced AMI in combination with a high-fat diet. Pharmacological activation of 5-HT7R alleviates disease-induced upregulation of creatine kinase and troponin-I, as well as tumor necrosis factor-α, interleukin-6, and transforming growth factor β1 expression. Cardiac tissue displayed less immune cell infiltration, reduced necrosis, and less hemorrhage after LP44 treatment. This improved tissue remodeling is regarded to be overall cardioprotective.^95^ Follow-up studies investigated the role of constitutive 5-HT7R activity during the regulation of the coronary flow but did not confirm its relevance.^93^

All these data suggest that activation of 5-HT7R signaling might prove beneficial after AMI. Therefore, on-the-market drugs with 5-HT7R-affinity should be screened for their therapeutic potential in future studies (see Table 2).

**Table 2:** Binding Affinities of Discussed Compounds^a^

Compound	5-HTR	Agonist (+)/Antagonist (−)	Binding affinities [p*K*_*i*_]	Off-target receptor	Binding affinities [p*K*_*i*_]	Agonist (+)/Antagonist (−)
5-HT	5-HT1AR^b^	(+)	9.1–9.7	D1 receptor	5	(+)
	5-HT1BR^b^	(+)	7.4–9.0	D5 receptor	5.5	(+)
	5-HT1DR^b^	(+)	8.0–9.0	Proton-coupled amino acid	2.2	Inhibitor
	5-HT1eR^b^	(+)	8.0–8.2	transporter 1		
	5-HT1FR^b^	(+)	7.7–8.0			
	5-HT2AR^b^	(+)	6.0–8.4			
	5-HT2BR^b^	(+)	7.9–8.4			
	5-HT2CR^b^	(+)	6.8–8.6			
	5-HT3R^b^	(+)	6.0–6.9			
	5-HT4R^b^	(+)	5.9–7.0			
	5-HT5AR^b^	(+)	6.7–6.9			
	5-HT6R^b^	(+)	6.8–7.5			
	5-HT7R^b^	(+)	8.1–9.6			
8-OH-DPAT	5-HT1AR^b^	(+)	8.4–9.4			
	5-HT1BR	(+)	6.2 [pIC_50_]			
	5-HT1DR	(+)	6.9–7.3			
	5-HT1eR	(+)	5.5			
	5-HT1FR	(+)	5.8			
	5-HT2AR	(+)	5.6			
	5-HT2BR	(+)	5.4			
	5-HT2CR	(+)	5.6			
	5-HT5AR	(+)	5.6–5.7			
	5-HT7R	(+)	6.3–7.6			
Flesinoxan	5-HT1AR	(+)	9.3			
Flibanserin	5-HT1AR^b^	(+)	9.0			
	5-HT2AR	(−)	7.3			
Ketanserin	5-HT1AR	(−)	5.0	D1 receptor	6.7	(−)
	5-HT1BR	(−)	5.2–5.4 [pIC_50_]	D5 receptor	5.6	(−)
	5-HT1DR	(−)	7.4–7.5	α1A-adrenoceptor	8.2	(−)
	5-HT2AR^b^	(−)	8.1–9.7	α1B-adrenoceptor	8.2	(−)
	5-HT2BR	(−)	6.1–6.7	α1D-adrenoceptor	7.8	(−)
	5-HT2CR	(−)	6.8–7.5	Vesicular monoamine transporter 1	5.8	Inhibitor
	5-HT5AR	(−)	4.7	Vesicular monoamine transporter 2	6.3	Inhibitor
	5-HT7R	(−)	5.9–6.5			
LP44	5-HT1AR	(+)	7.3	D2 receptor	8.1	(+)
	5-HT2AR	(+)	6.5			
	5-HT7R^b^	(+)	9.7			
LY-272015^156^	5-HT2AR	(−)	7.65 (rat)			
	5-HT2BR	(−)	9.86 [p*K*_b_] (rat)			
	5-HT2CR	(−)	7.92 (mouse)			
Piboserod (SB207266)	5-HT2BR	(−)	6.3–6.6			
	5-HT4R^b^	(−)	8.8–10.4			
Sarpogrelate	5-HT2AR	(−)	8.5			
	5-HT2BR	(−)	6.6			
	5-HT2CR	(−)	7.4			
SB200646^157^	5-HT2AR	(−)	<5.0			
	5-HT2BR^b^	(−)	6.2			
	5-HT2CR^b^	(−)	6.3			
SB204741	5-HT2BR^b^	(−)	6.9			
	5-HT2CR	(−)	5.6			
SB269970	5-HT7R	(−)	8.6–8.9			
Terguride	5-HT1AR	(+)	8.5	D2 receptor^b^	9.1	(+)
	5-HT1BR	(+)	6.6	D2 receptor^b^	8.9	(−)
	5-HT1DR	(+)	7.8	D3 receptor	9.0	(+)
	5-HT2AR^b^	(−)	8.3	D4 receptor	8.1	(−)
	5-HT2BR^b^	(−)	8.2	α1A-adrenoceptor	8.5	(−)
	5-HT2CR	(−)	7.3	α2A-adrenoceptor	9.5	(−)
				α2B-adrenoceptor	9.4	(−)
				α2C-adrenoceptor	9.1	(−)

a All listed binding affinities were determined for human receptors, unless otherwise noted. Symbols used: (+) agonist, (−) antagonist, and D: dopamine. Data extracted from IUPHSR/BPS Guide to pharmacology database,^155^ unless otherwise noted.

b Main target receptor.

#### Serotonin Receptors and the Immune Response After AMI

Additionally to the direct effects of 5-HT on the cardiac tissue via therein expressed serotonin receptors (see Figure 3), 5-HT also regulates functions of immune cells. This could also be important in the case of AMI, because after AMI, multiple species of immune cells expressing different 5-HT receptors infiltrate into the damaged area (see Table 1 and Figure 1).

Neutrophils are the first cells to infiltrate the infarcted tissue within the first 72 h and to initiate inflammatory responses.^158^ In TPH1 null mice, which do not express peripheral 5-HT, reduced infiltration of neutrophils into the infarct area was observed, with beneficial outcome.^26,159^ However, a prior study provided contradictory results demonstrating no effect of 5-HT on neutrophil migration *in vitro*.^160^ The expression pattern of 5-HTRs and the impact of 5-HT on neutrophil functions thus needs further investigation.

Monocytes and macrophages play a pivotal role in the acute immune response within the cardiac tissue as well as for the tissue remodeling and mitigation of inflammation in the subacute phase after AMI. Therefore, we will focus in the following text on the possible contribution of 5-HTRs expressed on these cells.

After AMI, monocytes and macrophages infiltrate the damaged myocardium and, together with tissue-resident macrophages, support the acute inflammatory response and also participate in the resolution of inflammation after about 1 week. This transition in the tissue between both phases is regarded to be critical for the overall outcome after AMI. Monocytes and macrophages were reported to express 5-HT1AR, 5-HT1ER, 5-HT2AR, 5-HT2BR, 5-HT2CR, 5-HT3R, 5-HT4R, and 5-HT7R.^49,161,162^ Depending on their environment, macrophages can be polarized toward pro- and anti-inflammatory phenotypes, and the differentiation stage determines their expression profile of 5-HTRs. Macrophages with an anti-inflammatory phenotype preferentially express 5-HT2BR and 5-HT7R. It has been shown that serotonin can facilitate the differentiation of macrophages toward an anti-inflammatory polarization, and this effect was mediated by stimulation of 5-HT2BR and 5-HT7R.^71^ On the other hand, inhibition of the 5-HT2AR by ketanserin was also shown to shift macrophage differentiation toward an anti-inflammatory phenotype and to promote macrophages’ anti-inflammatory properties.^54^

Phagocytosis is one of the key properties of macrophages, and 5-HT stimulation was shown to enhance phagocytosis in mouse peritoneal macrophages in a 5-HT1AR-dependent manner.^163^ In mouse bone-marrow-derived macrophages, 5-HT stimulation modulated phagocytosis depending on interferon gamma (IFN-γ) concentration, augmenting phagocytosis at low IFN-γ levels, and reducing antigen-presenting capacity of macrophages.^164^ IFN-γ was later shown to downregulate phagocytosis in macrophages via the PI3K-Akt-mTor pathway, which is also a signaling axis of several 5-HTRs, including 5-HT1R and 5-HT2R.^165^ Another important regulator of phagocytosis is the RhoA/Rho Kinase (ROCK) signaling pathway, which can be activated by 5-HT4R-Gα13 signaling. Activation of this signaling pathway has been shown to reduce phagocytic engulfment in murine macrophages.^166^

Moreover, 5-HT treatment changed the cytokine and chemokine release profile of human monocytes, most probably via activation of 5-HT3R, 5-HT4R, and 5-HT7R.^49^ Also human macrophages reduced their pro-inflammatory cytokine secretion and acquired an anti-inflammatory profile upon 5-HT treatment.^167^

Additional crucial players during the initiation of the tissue remodeling process after AMI are dendritic cells (DCs). Following AMI, DCs migrate into the border zone of the infarct region. Their numbers peak at day 7 post AMI.^168^ Their presence is impacting the infiltration of other immune cells: Ablation of CD11c^+^-DCs resulted in enhanced tissue infiltration of pro-inflammatory macrophages in a mouse model of AMI.^168^ Contrary, depletion of CD103^+^/CD11b^+^-DCs was shown to reduce immune cell infiltration and improve heart functions.^169^ During their maturation process, DCs shift their 5-HTR expression profile: immature DCs were shown to express 5-HT1BR, 5-HT1ER, and 5-HT2BR, while mature DCs express 5-HT4R and 5-HT7R. Both, immature and mature DCs, express the 5-HT2AR and 5-HT3R.^170^ Activation of the 5-HT3R, 5-HT4R, and 5-HT7R shifted the secretion profile of DCs toward anti-inflammatory signaling. This might mitigate an excessive pro-inflammatory reaction and support tissue remodeling.^170,171^ 5-HT can further govern oriented migration of immature DCs via stimulation of 5-HT1R- and 5-HT2R-mediated signaling.^171^ Moreover, in mature DCs, activation of the 5-HT7R enhanced chemotactic motility toward a chemokine (C–C motif) ligand 19-gradient *in vitro*.^172^

DCs bridge the innate with the adaptive immune response by priming of T lymphocytes. Noteworthy, 5-HT stimulated DCs express less costimulatory molecules, which results in reduced activation of (allogenic) T cells via 5-HT1R and 5-HT7R signaling.^173^ Cross-priming DCs were additionally able to stimulate CD4^+^ as well as CD8^+^ T cells. This resulted in the initiation of a persistent inflammatory response following AMI.^174^

In addition, 5-HT can act on T cells via direct stimulation of multiple 5-HTRs expressed on these cells (see Figure 1). Pharmacological inhibition of 5-HT1AR and 5-HT7R dampens T cell proliferation.^45,175,176^ Ablation of T cells using a recombination activating gene 1 (RAG1) null mouse resulted in a smaller scar region after AMI, which was mainly mediated by an alleviated response of CD4^+^ T cells.^177^ Treatment with the SSRI fluoxetine in rats elevated the number of T cells expressing 5-HTT and decreased the fraction of CD4^+^ while increasing the fraction of CD8^+^ T cells.^178^ This might contribute to the positive effects of SSRI treatment on cardiovascular events.

Another important component of the adaptive immune response are B cells. They influence the tissue healing process after AMI through production of antibodies. Elevated levels of 5-HT in the blood, as observed after AMI, mainly impact proliferation and thereby activation of B cells. Among others, mitogen-stimulated proliferation has been shown to be enhanced through 5-HT via 5-HT1AR signaling.^31^ Stimulation with 5-HT or SSRIs treatment with fenfluramine resulted in controlled B cell death and apoptosis.^179−181^ However, most of these investigations were carried out in lymphoma cells. In the context of AMI, where B cells peak at day 7, the 5-HT system might enhance proliferation and therefore result in an adverse outcome.^158,182^

Since elevated levels of 5-HT are often detected in the heart after AMI, it can be assumed that serotonergic signaling is generally enhanced due to increased activation of 5-HTRs expressed in various cell types, also including the mentioned immune cells. Therefore, the targeted modulation of defined 5-HTRs on the invading immune cells represents an interesting therapeutic option to improve the wound healing process after AMI.

## Conclusions

Given their highly diverse signaling actions, there is no explicit answer to the question if serotonin receptors in AMI can be regarded as friends or foes. Therefore, each receptor has to be assessed individually. Positive effects on outcome after AMI could be conceivable by activation of 5-HT1R and 5-HT7R (friends?) or by inhibition of the 5-HT2R and 5-HT4R (foes?). Selective targeting of single families of serotonin receptors seems inevitable. Since these receptors are expressed as multiple isoforms and engage in several G protein-mediated as well as G protein-independent signaling pathways, receptor-specific signaling-biased compounds might be necessary to evoke desirable action and reduce unfavorable side-effects.

Another challenge of targeting 5-HTRs for clinical application is the fact that serotonin receptors can form heterodimers with other GPCRs and even non-GPCRs. For example, our study demonstrated the existence and functional relevance of 5-HT1AR/5-HT7R heterodimers.^183^ The 5-HT4R has been shown to interact with the 5-HT2AR and the histamine receptor, both of which are also present in cardiac tissue.^184,185^ More recently, we demonstrated the physical interaction between 5-HT2AR and tropomyosin receptor kinase B as well as 5-HT7R and CD44.^186,187^ Therefore, pharmacological modulation of selective 5-HTRs might subsequently change signaling of their interaction partners.

Undoubtedly, the 5-HT system is impacted by AMI and represents a link in the connection of depressive behavior and cardiac disease on the heart-brain axis. Recent evidence support the view that the serotonergic system is an important component mediating organ injury after AMI, especially by modulation of the inflammatory response in the wound healing process.^188^ Thus, targeting specific serotonin receptors to improve functional outcome after AMI with the overall goal to improve quality of life represents a valuable objective for future studies.

## Abbreviations

5-HT: 5-hydroxytryptamine
5-HT system: serotonergic system
5-HTR: serotonin receptor
5-HTT: serotonin transporter
8-OH-DPAT: 8-hydroxy-2-(di-N-propylamino)tetralin
AC: adenylyl cyclase
AMI: acute myocardial infarction
CamKII: calmodulin-dependent protein kinase II
Cdc42: cell division control protein 42
CDK5: cyclin-dependent kinase 5
CNS: central nervous system
CREB: cAMP response element-binding protein
D: dopamine
DC: dendritic cells
DOCA: deoxycorticosterone acetate
EF: ejection fraction
ERK: extracellular-signal regulated kinase
flow: flow cytometry
GI: gastrointestinal
GIRK: G protein-coupled inwardly rectifying potassium channel
GPCR: G protein-coupled receptor
IFN-γ: interferon gamma
IHC: immunohistochemistry
IP3: inositol 1,4,5-trisphosphate
LV: left ventricle
LVEF: left ventricular ejection fraction
m: mouse
MAPK: mitogen-activated protein kinase
MDD: major depressive disorder
mTOR: mammalian target of rapamycin
NF-kB: nuclear factor “kappa-light-chain-enhancer” of activated B-cells
PDE: phosphodiesterase
PI3K: phosphoinositide-3-kinase
PKA: protein kinase A
PKB/Akt: protein kinase B
PKC: protein kinase C
PLC: phospholipase C
RAG1: recombination activating gene 1
RhoA: ras homolog family member A
ROCK: RhoA/Rho Kinase
SERT: serotonin transporters
SRE: serum response element
SSRI: selective serotonin reuptake inhibitor
stim: pharmacological treatment
TCA: tricyclic antidepressants
TPH: tryptophan hydroxylase
TRPM8: transient receptor potential cation channel subfamily M member 8
